# Emergence and Control of Viral Respiratory Diseases

**DOI:** 10.3201/eid1104.050076

**Published:** 2005-04

**Authors:** Robert Webster, Stanley Plotkin, Betty Dodet

**Affiliations:** * St. Jude Children's Research Hospital, Memphis, Tennessee, USA;; † University of Pennsylvania, Philadelphia, Pennsylvania, USA;; ‡ Dodet Bioscience, Lyon, France

**Keywords:** zoonoses, attributes, commentary

**Emergence and Control of Viral Respiratory Diseases, a symposium**

Les Pensières, Veyrier du Lac, France 

November 30–December 3, 2004

Pandemic influenza is imminent and severe acute respiratory syndrome (SARS) may reappear. In addition, well-characterized diseases may reemerge through accidental or deliberate release of biologic agents. New diseases are likely to emerge at an accelerated frequency because precipitating factors already exist. Increased surveillance and preparedness are keys for prevention and control. These were the predominant conclusions of delegates from Europe, America, and Asia who met at the symposium on "Emergence and Control of Viral Respiratory Diseases," organized by the Merieux Foundation with the support of Sanofi Pasteur, November 30–December 3, 2004, at Les Pensières, Veyrier du Lac, near Annecy, France (in the French Alps). Participants assessed the role of ecologic, viral, and host factors, as well as strategies for prevention and control through surveillance, vaccination, and treatment.

Many factors contribute to the ability of viruses to cross species and their dissemination in humans. Fifty percent of known human pathogens and nearly 75% of all emerging infectious diseases are zoonotic in origin. Many of these pathogens have spilled over from natural wildlife reservoirs into human populations, either directly or through contact with domestic or peridomestic animals. Viruses evolve quickly, and many disease-causing agents already exist in nature. Pathogens jump species often, in spite of "species barriers," which vary from pathogen to pathogen. For viruses, the barrier may include receptor specificity, the need for coreceptors, different favorable temperature conditions, host-specific transcription factors, or innate immunity. However, modern conditions provide ever greater opportunities for viruses to cross species. An increase in world population is driving a need to develop new territories and to find new sources of food and water. More species are being raised or hunted for food, thus contributing to a juxtaposition of animal and human populations (Steven S. Morse, Columbia University, New York, NY, USA, and Jonathan Epstein, Consortium for Conservation Medicine, Palisades, NY, USA).

Respiratory pathogens can be transmitted through air and water. Fungal spores can be transported globally in clouds of desert dust. Human wastewater is affecting coastal water quality on a global scale. Globally, ≈90% of sewage generated in coastal environments is released untreated, and the wastewater contains high numbers of fecal-oral pathogens, particularly viruses, which can survive for extended periods. The wastewater-associated viruses can cause a variety of illnesses, including gastroenteritis, ocular infections, hepatitis, myocarditis, and respiratory diseases. (Dale Griffin, U.S. Geological Survey, St. Petersburg, FL, USA). In zoonotic infections, although many interspecies transfers are dead ends, sometimes viruses evolve through mutation, recombination, or reassortment and acquire the potential to spread from human to human. RNA viruses, which lack proof mechanisms to ensure fidelity of the genome, accumulate mutations during replication and are the most able to adapt to new hosts. However, RNA viruses have diverse evolutionary histories. The evolutionary trajectories of measles, caused by a morbillivirus that emerged 2,000–5,000 years ago, and AIDS, caused by a retrovirus (HIV-1) that originated in the 20th century, have been quite different. The pandemic form of HIV-1, group M, originated around 1930 and has diversified through rapid mutation and recombination, whereas measles viruses evolve ≈10 times more slowly (Paul Sharp, University of Nottingham, Nottingham, UK).

Human activities, including migration and travel, may disseminate a localized outbreak. During the 1990s, >5,000 airports had regularly scheduled international flights, and ≈2 million persons crossed international borders daily. The World Tourism Organization anticipates 1.6 billion international tourists by 2020 (Mary Wilson, Harvard School of Public Health, Boston, MA, USA).

## Surveillance and Control

Strategies for dealing with emerging diseases include giving special attention to situations promoting emergence (especially at the animal-human interface), effective surveillance (early warning and identification), and control. Global warning systems have been set up, such as ProMED mail, initiated over 10 years ago, and the World Health Organization (WHO) Global Outbreak Alert and Response Network. By compiling a comprehensive database of human pathogens that have emerged within the past 50 years, researchers have identified the factors that have most frequently contributed to pathogen emergence or reemergence. From the analysis of common characteristics, "hotspots" of disease emergence can be defined, allowing regions that may be at particular risk to be identified (S. Morse, J. Epstein).

After an outbreak has been identified, the pathogenic agent must be characterized. The classic laboratory methods (cell culture, electron microscopy, immunodetection) largely contribute to viral detection and routine diagnosis. Modern polymerase chain reaction (PCR) technology facilitates rapid detection of viruses. However, PCR inhibitors are frequently encountered in severely ill patients or in inappropriate specimens. Appropriate virus concentrations may be found only in body sites from which patient samples are difficult to obtain. SARS-coronavirus (SARS-CoV) is best detected from sputum or endotracheal aspirates, but sampling poses a severe risk for aerosolization of infectious particles. The overall sensitivity in mixed samples from acutely ill patients is only ≈70%; thus SARS cannot be ruled out by a negative reverse transcription–PCR result (Christian Drosten, Bernhard-Nocht, Institute for Tropical Medicine, Hamburg, Germany).

New techniques such as gene chips/biosensors or VIDISCA (Virus Discovery-cDNA-AFLP) method are valuable for detecting unknown viruses (Lia van der Hoek, University of Amsterdam, Amsterdam, the Netherlands). The latter method has helped identify a fourth human coronavirus (in addition to HCoV-229E, HCoV-OC43, and SARS-CoV); HCoV-NL63 was isolated from a 7-month-old child with bronchiolitis and conjunctivitis, and its sequence analysis indicates that it has been circulating in humans for >15 years (A.D.M.E. Osterhaus, Erasmus Medical Center, Rotterdam, the Netherlands). In fact, to accelerate the identification of a new pathogen, both new and classic methods are essential. Experience gained during the 2003 SARS outbreak showed the importance of a close relationship between microbiologists and pathologists. Histopathologic examination was essential in identifying virally infected cells in tissue sections through the use of appropriate antibodies in immunohistochemical tests, and in situ hybridization techniques were crucial in advancing knowledge of this new infection. Electron microscopy helped identify the novel virus, and tissue sections from biopsy and autopsy specimens showed the underlying SARS pathogenesis.

However, few pathologists are well trained in virology, especially in many parts of the Asian-Pacific region, the epicenter of possible future outbreaks (4 pathologists/million are found in Asia versus 100/million in the USA); and because of Asian cultural values, obtaining autopsy or biopsy tissue from infected patients is limited. Therefore, the number of regional centers where advanced molecular and diagnostic testing can be performed should be increased, and the links between pathologists (veterinary and human) and virologists should be improved (John Nicholls, University of Hong Kong, Hong Kong, Special Administrative Region [SAR], China).

## Lessons from the SARS Outbreak

SARS, which first emerged in southern China in November 2002, provided a painful reminder of the global impact of emerging infectious diseases. Large, centralized marketplaces proved to be venues for transmissions to humans. Spread occurred in hospitals, and a local outbreak became a potential pandemic through international travel. By July 5, 2003, when the epidemic was interrupted, 8,099 cases had occurred (21% among healthcare workers) with 774 deaths (Wilina Lim, Centre for Health Protection, Hong Kong, SAR, China). Four more cases (with 1 death) occurred in early 2004, illustrating the risk for accidental contamination of laboratory workers.

SARS-CoV is phylogenetically distinct from all previously known human and animal coronaviruses. The early index cases of SARS occurred independently, with scattered but restricted geographic distribution; the infection initially caused mild symptoms and little or no human-to-human infectivity. SARS-CoV evolved toward greater fitness with the human host during the outbreak. Major genetic variations in some critical genes, particularly the spike gene, seem to have been essential for transition to human-to-human transmission (Guoping Zhao, Chinese National Human Genome Centre, Shanghai, China).

The speed of the scientific response to this new viral disease was unparalleled. The clinical syndrome was described, the etiologic agent identified, diagnostic tests devised, and the genome completely sequenced within weeks. However, the global success in containing SARS owed much to traditional public health methods of clinical case identification, contact investigation, patient isolation, and quarantine. (Stanley Plotkin, University of Pennsylvania, Philadelphia, PA, USA). However, should a second global SARS outbreak occur, clinicians would not have controlled data on which to base therapeutic decisions (Susan Poutanen, Mount Sinai Hospital, Toronto, Ontario, Canada). In the initial phases of the SARS outbreak, patients received empiric treatments, mainly ribavirin (since it is active against a broad range of RNA and DNA viruses), and corticosteroids (since it was suggested that lung injury could result from immunopathogenic mechanisms). Other treatments, including passive immunization with serum specimens from convalescent-phase patients and type 1 interferons, were used to treat SARS patients, with anecdotal success. But no controlled clinical trial was conducted.

The unpredictable nature of outbreaks poses many challenges to the successful design and implementation of controlled trials, but we must be prepared to conduct such trials if SARS reemerges or future outbreaks of novel pathogens occur (S. Poutanen). National and international collaborative groups should be created, supported by appropriate funding and with a mandate to implement clinical trials of therapeutic agents in outbreak settings. The collaboration of ethics committees is critical in establishing a process that facilitates trials, while ensuring the safety of participating patients, researchers, and communities.

Key to the development of effective antiviral drugs and vaccines against SARS-CoV is the availability of animal models. A variety of animal species are susceptible to infection with SARS CoV, including rodents (mice, rats, and hamsters), nonhuman primates (cynomolgous, rhesus, and African green monkeys), ferrets, and cats. While notable pathologic changes develop in the lungs of ferrets and hamsters, infected mice showed no clinical signs of disease and only mild inflammation, although extensive virus replication occurs, peaking 3–4 days after infection. SARS-like symptoms and tissue damage typical of SARS in humans develops in experimentally infected cynomolgus macaques (Bart Haagmans, Erasmus Medical Center). This model was used by Albert Osterhaus' group to confirm the etiologic role of the newly identified CoV in SARS (it fulfilled Koch's postulates) and exclude the role of the metapneumovirus.

Various antiviral agents have been tested in vitro and in animal models, including ribavirin, protease inhibitors, receptor-binding inhibitors, virus-cell fusion inhibitors, and nitric oxide inducers and miscellaneous compounds such as chloroquine (Erik de Clercq, University of Leuven, Leuven, Belgium). Prophylactic treatment of SARS-CoV–infected macaques with pegylated recombinant interferon-a reduced viral replication and excretion as well as pulmonary damage.

## SARS Vaccines

A number of SARS candidate vaccines are being developed with a variety of approaches and technologies (B. Haagmans; Luis Enjuanes, National Biotechnology Centre, Barcelona, Spain; Gary Nabel, National Institutes of Health, Vaccine Research Center, Bethesda, Maryland, USA). Whole inactivated, live attenuated viruses, DNA vaccines, genetically engineered viral vectors, replication-defective pseudovirions or pseudoparticles expressing SARS proteins are being tested with or without adjuvants, in prime-boost schedules and by intramuscular or intranasal routes. An inactivated vaccine has completed phase 1 evaluation, and preliminary results indicate that it was safe and well tolerated. Other phase 1 clinical trials have just started or are imminent.

Without SARS-CoV circulating in the population (and it is hoped this situation will continue), however, a main challenges is to complete the clinical evaluation of efficacy of candidate vaccines. Given the differences observed in SARS-CoV pathogenesis in different animal species and in humans, candidate vaccines may need to be evaluated in several SARS animal models.

Immunopathogenic mechanisms may, in fact, be triggered by a SARS vaccine; candidate vaccines designed to protect cats against a feline coronavirus actually accelerate disease and death from the virus. However, no current evidence indicates that SARS vaccines are capable of enhancing disease in animal models. On the other hand, a recombinant modified vaccinia virus Ankara (rMVA) expressed the SARS-CoV spike (S) protein, which induced a rapid and vigorous neutralizing antibody response after challenge with SARS-CoV in mice and ferrets, also led to a strong inflammatory response in liver tissue of ferrets (B. Haagmans).

## Influenza Pandemic: Surveillance, Preparedness, and International Cooperation

Pandemic influenza may arise by the transmission of influenza A viruses or virus gene segments from aquatic bird reservoirs to humans and domestic animals. Of the 16 hemagglutinin subtypes of influenza identified in nature, only H1, H2, and H3 subtypes have caused pandemics and epidemics in humans. An H1N1 virus was at the origin of the 1918–1919 pandemic that killed 40 million people globally. H2 viruses were at the origin of the 1957–1958 pandemic, and H3 viruses entered the human population in 1968, causing the Hong Kong pandemic. Although H1 and H3 viruses are still circulating in humans (after undergoing antigenic drifts), H2 viruses disappeared in 1968; however, they are still circulating in wild aquatic birds, and they exist in laboratories. The risk for accidental release should not be underestimated (Robert Webster, St Jude Children's Hospital, Memphis, TN, USA). The severe and localized outbreaks of A (H3N2) influenza in Madagascar in August 2002 and in the Democratic Republic of Congo in November of that year demonstrated the extent of this disease in nonindustrialized tropical countries and underlined the need to improve the understanding of the health and economic effects of influenza in the world, particularly in regions such as Africa (Jean-Claude Manuguerra, Institut Pasteur, Paris, France).

Other subtypes such as H5, H7, and H9 also have a high pandemic potential. Although so far they have not spread continuously from person to person, they have infected humans who have had contact with birds, in whom the viruses circulate. H7 viruses infected poultry and humans in the Netherlands (H7N7) and in British Columbia (H7N3). The culling of 19 million domestic birds in British Columbia has eradicated the virus from commercial poultry. H9 viruses were first isolated in 1999 in 2 young children hospitalized in Hong Kong. These viruses are endemic in poultry in Eurasia, continue to reassort with other as yet unidentified influenza viruses, and maintain the human receptor specificity (R. Webster).

The highest concern relates to the H5N1 influenza virus, which is widely entrenched in Asia, where it is circulating in a wide variety of avian species. It first emerged in Hong Kong in 1997, when 18 people were infected and 6 died of the disease. Approximately 2 million domestic birds were culled; the outbreak was stopped and the virus eradicated, but the parental virus still circulated in geese and ducks in coastal China and reemerged in 2003 to 2004. By November 2004, 44 human cases of avian influenza had been identified, causing 32 deaths.

These events show that receptor specificity is not an absolute barrier to infection. Avian H5N1 viruses were transmitted directly from infected poultry to humans on ≤18 and likely >40 occasions in 1997 and 2003–2004, respectively, despite H5N1 retention of avian receptor specificity. None of these strains was easily transmitted from person to person. However, H5N1 viruses have the potential to ignite the next pandemic, should they acquire the capacity to be transmitted from person to person, either through mutation or reassortment with human influenza viruses.

The H5N1 viruses that caused the initial human outbreak in Hong Kong in 1997 and subsequent avian outbreaks in 2001 and 2002 evolved through a series of genetic reassortments, which gave rise to a dominant H5N1 genotype in chickens and ducks that was responsible for the regional outbreak in 2003 to 2004. Domestic ducks had a central role in the generation and maintenance of this virus, while wild birds may have contributed to the increasing distribution of the virus in Asia. Furthermore, both the 1997 and 2003 human H5N1 isolates acquired the ability to induce an overproduction of pro-inflammatory cytokines by primary human macrophages in vitro (which could explain their pathogenicity) (Leo Poon, University of Hong Kong).

WHO takes the threat very seriously and has defined prevention and control of influenza pandemics and epidemics as a top priority. For over 50 years, WHO has been coordinating an international program for influenza virus surveillance. An influenza pandemic preparedness plan was drawn up in 1999, which defines the responsibilities of WHO and national authorities in case of an influenza pandemic and strongly recommends that all countries establish and implement national pandemic plans. In addition, a global agenda on influenza was set up in 2002 to provide impartial guidance on research, development, and global action for influenza control, and also to support the coordination of actions for influenza control and surveillance.

Some tasks have been completed, but the preparation for the next pandemic is woefully inadequate (David Fedson, Sergy Haut, France). Although a pandemic threat will likely be recognized rapidly and the virus isolated and characterized within a few weeks, it may take months to produce a specific pandemic vaccine. The neuraminidase inhibitors (zanamivir and oseltamivir), which have been licensed for the prophylaxis and treatment of influenza virus infections, are likely to be effective against new emerging influenza viruses. But preventing the emergence of a new pandemic with antiviral agents requires stockpiling large quantities and planning for targeted prophylaxis.

Producing an adequate supply of pandemic vaccine and ensuring its equitable distribution to all countries represents one of the most compelling challenges facing global public health. Producing the annual trivalent vaccines against influenza in embryonated chicken eggs currently takes 6 months. To respond to a pandemic, vaccines with a high efficacy will have to be produced more rapidly and more flexibly. Current developments in influenza vaccine preparation include novel production technologies (e.g., production in cell culture), reverse genetics technology for the generation of vaccine strains, novel adjuvants to improve vaccine immunogenicity, and alternative routes of antigen administration (Ron Fouchier, Erasmus Medical Center).

Preparedness, moreover, implies solving the intellectual property issues for using reverse genetics, overcoming regulatory concerns about using genetically modified organisms, conducting international publicly funded clinical trials of candidate vaccines, developing an international process for rapid vaccine registration, defining responsibility for pandemic vaccine liability, expanding interpandemic vaccine use, forecasting vaccine demand in each country, and overcoming political obstacles to distributing vaccine to "have not" countries (D. Fedson).

One of the key elements of the successful containment of emerging diseases is early detection and the ability to mount a coordinated global response. The WHO Global Outbreak Alert and Response Network plays the major role in this coordination. The network brings together ≈20 partner institutions throughout the world and focuses global resources. From 2000 to 2004, WHO assistance was requested for 62 outbreaks. From 2001 to 2003, 636 events were detected in 136 countries; 482 (78.5%) were verified, while 13.5% were unverifiable and 10.7% unsubstantiated (May Chu, WHO, Geneva, Switzerland).

Increasing zoonotic pressures associated with a growing human population and rising affluence in Southeast Asia raise the likelihood of new and reemerging infectious diseases. The demand for animal protein in the diet has resulted in continuing increases in the population of pigs, chickens, and ducks in high-density farming operations (both in Asia and the United States). These operations are frequently developed without adequate biosecurity, which puts them in direct contact with wildlife, creating supersized amplifiers for virus evolution. Improving preparedness and response is a global necessity.

